# The Effectiveness of Psychological Interventions for Patients Undergoing Anterior Cruciate Ligament Reconstruction: A Meta-Analysis and Systematic Review

**DOI:** 10.3390/jcm15103980

**Published:** 2026-05-21

**Authors:** Manxue Zhang, Bohua Li, Jialiang Tian, Yi Huang, Xiaobing Pu

**Affiliations:** 1 Child Mental Health Center, National Center for Mental Disorders, West China Hospital, Sichuan University, Chengdu 610207, China; 2 Laboratory of Child and Adolescent Psychiatry, Mental Health Center and Psychiatric Laboratory, West China Hospital, Sichuan University, Chengdu 610207, China; 3 Department of Orthopedic Surgery and Orthopedic Research Institute, West China School of Public Health and West China Fourth Hospital, Sichuan University, Chengdu 610207, China

**Keywords:** anterior cruciate ligament, psychological interventions, knee arthroscopy, randomized controlled trial, clinical outcomes, meta-analysis

## Abstract

**Background**: No systematic review has yet been conducted simultaneously on the effectiveness of psychological interventions across multiple outcome measures during rehabilitation following anterior cruciate ligament reconstruction (ACLR). This study aims to assess the effects of such interventions on pain, psychological outcomes, patient-reported knee function, objective knee measures, and quality of life following ACLR. **Methods**: We searched PubMed, Medline, Embase, PsycINFO, and the Cochrane Library from inception to 20 April 2026 (PROSPERO CRD42023483889). Eligible randomized controlled trials compared psychological interventions with usual care in ACLR patients. Two reviewers assessed eligibility, risk of bias, and extracted data. Random-effects models were used; effect sizes were interpreted using Cohen’s guidelines. **Results**: Of 401 records screened, 11 RCTs (440 participants) were included. Psychological interventions significantly improved pain (six trials, SMD = −0.96, 95% CI −1.40 to −0.52, *p* < 0.001, I^2^ = 47%; large effect), kinesiophobia (TSK-11: five trials, SMD = −0.48, −0.74 to −0.22, I^2^ = 0%; small effect), knee self-efficacy (K-SES: three trials, SMD = 0.53, 0.19–0.86, I^2^ = 0%, moderate effect), patient-reported knee function (IKDC: two trials, SMD = 0.58, 0.26–0.90, I^2^ = 0%, moderate effect), and physical role function (SF-36: two trials, SMD = 0.41, 0.04–0.78, I^2^ = 0%, small effect). No significant effects were found for KT1000, knee strength, SF-36 mental well-being, or ACL-RSI (all *p* > 0.05, with substantial heterogeneity for ACL-RSI). Particularly, imagery therapy reduced pain (three trials, SMD = −1.54, I^2^ = 15%). **Conclusions**: This meta-analysis provides preliminary evidence that psychological interventions, especially imagery therapy, may improve pain, psychological outcomes, patient-reported knee function, and quality of life after ACLR. Adequately powered trials with standardized protocols are needed to confirm these findings.

## 1. Introduction

The anterior cruciate ligament (ACL) is vital for knee stability, with ruptures being a common injury affecting 35 per 100,000 people annually [1]. These injuries are prevalent across age groups [2,3], increasing the risk of osteoarthritis later in life [4,5]. ACL reconstruction (ACLR) is the primary treatment [6], followed by rehabilitation to restore the pre-injury functional level [7]. However, outcomes are suboptimal, with 55–83% returning to pre-injury sports [8,9], and 3.4–18% experiencing re-rupture [9,10,11]. Recovery is influenced by physical and psychological factors [12,13]. Traditionally, rehabilitation has focused primarily on physical aspects, often overlooking psychological factors. Recent research has increased focus on the psychological aspects of rehabilitation [14,15,16,17].

ACLR patients face psychological challenges, such as the fear of re-injury [12,13], anxiety [18], depression [18], and decreased self-efficacy [19,20], affecting rehabilitation and return to sport [21,22]. Chronic pain triggers fear avoidance [23], while prolonged inactivity leads to mood disturbances and social disruption [18,21,22]. Psychological interventions in this study refer to non-pharmacological interventions that improve patients’ psychological states through psychological theories and techniques, thereby influencing the rehabilitation outcomes. These include, but are not limited to, cognitive-behavioral therapy, mindfulness-based stress reduction, imagery therapy, supportive psychotherapy, and psychoeducation, excluding mere health education or routine rehabilitation guidance [24]. They aid pain coping, reduce fear of re-injury, increase self-efficacy, foster a positive mindset [12,17,25,26], enhance rehabilitation motivation, and improve adherence, thereby potentially leading to better clinical results and faster recovery [27,28].

Psychological interventions like relaxation, guided imagery, modeling videos, exposure therapy, positive self-talk, goal setting, counseling sessions, and emotional disclosure have shown varying effectiveness in pain management, stress reduction, and recovery after ACLR [18,27,29,30,31,32,33,34,35,36,37,38]. Previous meta-analyses and systematic reviews have explored psychological aspects of ACLR rehabilitation, yet their scope remains limited. One focused on specific aspects like reducing kinesiophobia [39], another solely on return-to-sport rates [40], and a third examined psychosocial interventions for a broader spectrum of musculoskeletal sports injuries [41]. Furthermore, narrative reviews by Coronado et al. and Christino et al. summarized the importance of psychological factors in ACL reconstruction rehabilitation without quantitative synthesis [27,37]. However, there remains a lack of comprehensive meta-analysis specifically examining the effect of psychological interventions on pain, psychological outcomes, patient-reported knee function, objective knee outcomes, and quality of life following ACLR. Therefore, a new meta-analysis is warranted to synthesize current evidence on the effectiveness of psychological interventions across multiple outcome domains in ACLR rehabilitation, providing clinicians with evidence-based guidance for optimizing treatment strategy.

This meta-analysis aims to synthesize evidence on psychological interventions in ACLR rehabilitation across multiple outcomes. Using the PICO framework, we examine whether these interventions improve pain, psychological outcomes, patient-reported knee function, objective knee outcomes, and quality of life compared to usual care. We hypothesize superior outcomes, and the findings will inform the rehabilitation strategies.

## 2. Materials and Methods

We conducted the systematic review following guidance from the Preferred Reporting Items for Systematic Reviews and Meta-Analyses (PRISMA) [42]. The PRISMA 2020 Checklist was depicted in Supplementary Material S1. The protocol was registered on PROSPERO (CRD42023483889).

### 2.1. Search Strategies

The systematic literature searches were conducted in PUBMED, Medline, Embase, PsycINFO, and Cochrane Library databases for RCTs. Searches were conducted from inception to 20 April 2026, restricted to English. The primary search terms included: (“Anterior Cruciate Ligament” OR “Anterior Cruciate Ligament Injuries” OR “Anterior Cruciate Ligament Tear” OR “Anterior Cruciate Ligament Reconstruction” OR “ACL Injuries” OR “ACL Tears”) AND (“Psychotherapy” OR “Psychosocial Intervention” OR “Psychotherapeutic Processes” OR “Psychological” OR “Psychology”). Search strategies used are provided in Supplementary Material S2.

### 2.2. Inclusion and Exclusion Criteria

The following four criteria were applied to determine the eligibility of studies for inclusion in the review. (I) Population: patients underwent ACLR and (II) Intervention: psychological interventions in this study refer to structured, non-pharmacological interventions that aim to improve patients’ psychological states through established psychological theories and techniques, thereby influencing rehabilitation outcomes. Eligible interventions include but are not limited to cognitive-behavioral therapy, mindfulness-based stress reduction, imagery/guided mental practice, supportive psychotherapy, and formal psychoeducation. Mere health education or routine rehabilitation guidance without a defined psychological therapeutic framework was excluded. Multimodal interventions were eligible if the psychological component was clearly defined and primary. (III) Control: active treatment or control treatment (e.g., standard care, placebo, or no treatment) and (IV) Outcomes: assessment of postoperative pain severity, knee function, and quality of life (no time limit placed on assessment duration/follow-up). Studies have reported that outcome measures can be classified as the following: (1) Pain: Numerical Rating Scale (NRS)and Visual Analog Scale (VAS); (2) Psychological outcomes: Photographic Series of Sports Activities for ACLR (PHOSA-ACLR), Tampa Scale of Kinesiophobia (TSK), Knee Self-Efficacy Scale (K-SES), Anterior Cruciate Ligament–Return to Sport After Injury questionnaire (ACL-RSI), the State–Trait Anxiety Inventory (STAI), the Brief Resilience Scale (BRS), Locus of Control (LOC); (3) Patient-reported knee function: Knee Injury and Osteoarthritis Outcome Score (KOOS), Knee Outcomes Survey–Sports Activities Scale (KOS-SAS), the Lower Extremity Functional Scale (LEFS), the International Knee Documentation Committee system (IKDC), Lysholm score; (4) Objective knee outcomes: range of motion (ROM), KT-1000, knee strength, electromyographic (EMG); (5) Quality of life: 36-item short-form health survey (SF-36) and crutch-use days; and (6) Study type: RCT.

The following conditions were considered exclusion criteria: dissertations or conference abstracts, review articles, study protocols, studies comprising non-randomized designs, quasi-randomized trials, and studies lacking adequate outcome metrics. Any discrepancies in data extraction were resolved by consulting a third reviewer.

### 2.3. Screening

All citations retrieved from the database searches were imported into EndNote X8, followed by the elimination of duplicate entries. Two authors worked independently to screen potentially eligible articles at the title, abstract, and full-text levels. Inter-rater agreement was excellent at both stages (title/abstract: κ = 0.89; full-text: κ = 0.94).

Following the screening phase, any divergences in the results were deliberated upon by the two primary investigators, with a third researcher serving as an impartial adjudicator to resolve conflicts.

### 2.4. Data Extraction

A designated investigator transcribed pertinent data onto a uniform abstraction sheet, after which a secondary reviewer verified the completed forms against the original source manuscripts. The captured dataset encompassed: details of studies such as author, year, design, country; cohort demographics, such as population size, age distribution, gender composition, inclusion/exclusion criteria; specifications of psychosocial interventions and control arms, such as intervention type, duration, content, format; outcome metrics; and follow-up time points. To ensure the homogeneity of outcome measures, this study prioritized data collected at the post-intervention endpoint. If post-intervention data was unavailable for a specific outcome, the closest time point (around one month after surgery in this study) was included. To evaluate inter-group disparities, means accompanied by standard deviations (SD) and 95% confidence intervals were retrieved at baseline, post-operative phases, and subsequent follow-up assessments.

For studies with missing summary statistics (e.g., standard deviations), we contacted the corresponding authors via email. If no response was received, we calculated missing SDs from standard errors, confidence intervals, or *p*-values using methods outlined in the Cochrane Handbook.

### 2.5. Risk of Bias and Reporting Standards

The risk of bias for each RCT was independently assessed by two reviewers using the revised Cochrane risk of bias tool for randomized trials (RoB 2.0) [43]. This tool evaluates five domains: (1) randomization process, (2) deviations from intended interventions, (3) missing outcome data, (4) measurement of the outcome, and (5) selection of the reported result. Judgments for each domain and an overall risk of bias judgment were made as “Low risk,” “Some concerns,” or “High risk.” Disagreements were resolved by consensus or consultation with a third reviewer.

The Grading of Recommendations Assessment, Development and Evaluation (GRADE) framework was subsequently applied to determine the certainty of evidence for key outcomes. For each outcome, the initial certainty level was set as “high” based on the randomized trial design. The certainty was then downgraded by one or two levels for serious or very serious limitations in five domains: risk of bias, imprecision, indirectness, inconsistency, and publication bias. The final certainty of evidence was classified as high, moderate, low, or very low.

### 2.6. Strategy for Data Synthesis

Meta-analysis was performed using R software (version 4.4.1) with the meta and metafor packages when ≥2 studies provided sufficient data on a comparable outcome. For all continuous outcomes, the treatment effect was pooled as the standardized mean difference (SMD) with its 95% confidence interval (CI). The SMD (Hedges’ g) was selected as the primary effect size metric to allow for the synthesis of outcomes measured on different scales. A narrative synthesis was provided for outcomes where quantitative pooling was not feasible.

Model Selection and Heterogeneity Assessment: Given the anticipated clinical and methodological heterogeneity among the included trials, a random-effects model was chosen for all meta-analyses. Specifically, the restricted maximum-likelihood (REML) estimator was used for this model, with study weights assigned by the inverse-variance method. This model assumes that the true effect size varies across studies and provides a more conservative and generalizable estimate of the overall effect. To test the robustness of the pooled estimates, a sensitivity analysis was performed for each outcome that included more than three studies using a leave-one-out approach.

Statistical heterogeneity was quantified using Cochran’s Q test (with a significance level of *p* < 0.10) and the I^2^ statistic [44]. The I^2^ values were interpreted as follows: 0% to 40% (might not be important), 30% to 60% (moderate heterogeneity), 50% to 90% (substantial heterogeneity), and 75% to 100% (considerable heterogeneity) [45]. If substantial heterogeneity was detected (I^2^ > 50%, or *p* < 0.10), focused descriptive synthesis of specific psychological interventions was performed, where feasible, to explore potential sources of variation.

## 3. Results

### 3.1. Study Selection and Characteristics of the Selected Studies

**Figure 1:**
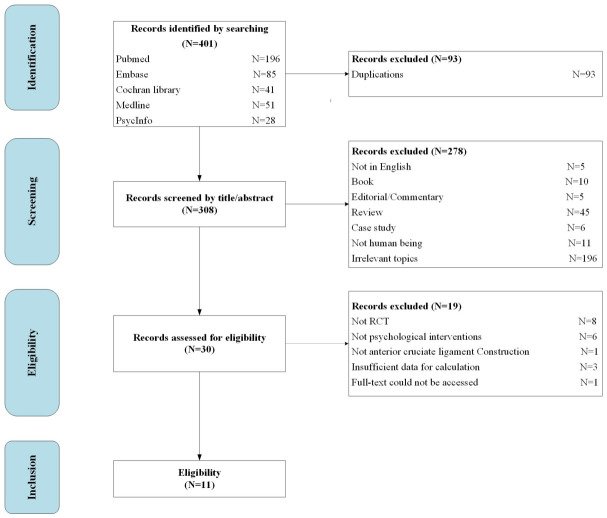
PRISMA flow diagram.

Systematic database searches initially identified 401 records. After removing duplicates and screening titles/abstracts, 30 full-text articles were assessed for eligibility. Of these, 11 RCTs met all inclusion criteria and were included in the meta-analysis [29,30,31,32,33,36,46,47,48,49,50]. A PRISMA flow diagram is provided in Figure 1. The eleven included articles were published between 2001 and 2026, and the sample sizes of each study ranged from 12 to 101. The general characteristics of RCTs included in the meta-analysis are shown in Table 1. Results were grouped into five broad categories based on the outcome used in the trial, including pain, psychological outcomes, patient-reported knee function, objective knee outcomes, and quality of life.

### 3.2. Quality Assessment and Risk of Bias

**Figure 2:**
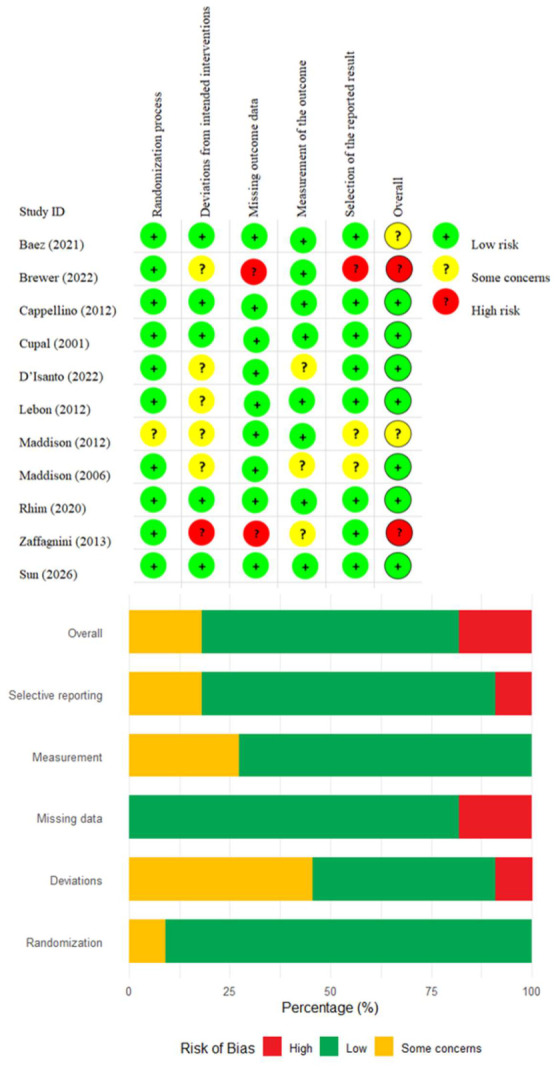
Risk-of-bias summary of included trials according to the RoB2 [29,30,31,32,33,36,46,47,48,49,50].

The RoB 2 and GRADE assessments for the 11 included RCTs are summarized in Table 2. Regarding the overall risk of bias (Figure 2), seven studies were rated as low risk, two as having some concerns [29,47], and two as high risk [32,36]. The main methodological concerns across studies were incomplete reporting of allocation concealment and randomization details, lack of blinding for participants and outcome assessors, and high or imbalanced attrition rates. The GRADE certainty of evidence ranged from moderate to very low, reflecting the aforementioned risk-of-bias issues and the pervasively small sample sizes. Specifically, five studies provided moderate-quality evidence, four provided low-quality evidence, and two provided very low-quality evidence [36,47]. The predominant reasons for downgrading were serious imprecision (small sample sizes and wide confidence intervals) and risk of bias (primarily absence of blinding). Notably, the very low-quality studies were early-phase exploratory trials with extremely small samples and unblinded subjective outcomes; their effect estimates should be interpreted with considerable caution in any quantitative synthesis.

### 3.3. Meta-Analysis of Pain

**Figure 3:**
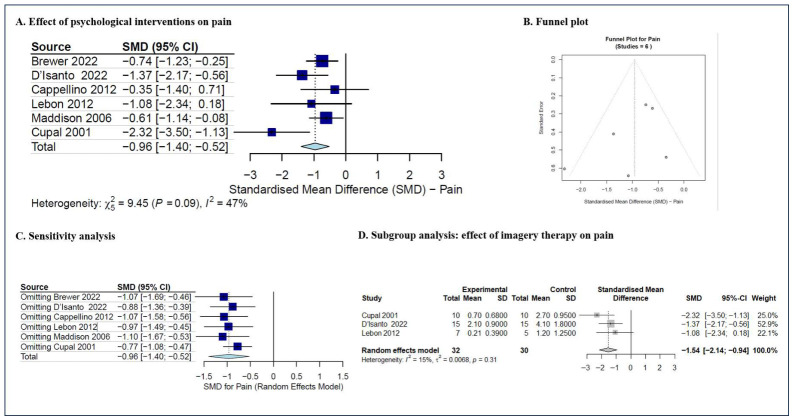
The meta-analysis of pain severity [30,33,36,46,48,49]. (**A**) The effect of psychological interventions on post-ACLR pain (SMD with 95% CI); (**B**) the funnel plot; (**C**) the sensitivity analysis; and (**D**) the effect of imagery therapy on post-ACLR pain (SMD with 95% CI).

Six studies collectively documented pain severity assessments for a cohort of 213 patients [30,33,36,46,48,49]. Pre- and postoperative pain were measured using the Visual Analog Scale (VAS) and the Numerical Rating Scale (NRS). The meta-analysis revealed that psychological interventions can reduce the pain compared to the non-psychological comparators among patients who underwent ACLR (six trials, SMD = −0.96, 95% CI (−1.40, −0.52), *p* < 0.001) with moderate heterogeneity (I^2^ = 47%, *p* = 0.09 for Cochran’s Q test) (Figure 3A). The predicted 95% CI was −1.88 to −0.08. Due to the small number of studies included, the funnel plot (Figure 3B) was generated for descriptive purposes only.

The sensitivity analysis revealed that the overall findings remained robust, as the sequential exclusion of individual studies did not induce substantial variations (Figure 3C). This stability underscores the reliability of our conclusions, even in the presence of observed heterogeneity.

Among the six trials reporting pain severity, three studies [30,46,48], with a total of 72 patients involved, adopted imagery therapy as the psychological intervention. Focused descriptive synthesis of these trials utilizing imagery therapy was depicted in Figure 3D. The results revealed that imagery therapy can reduce pain severity compared to the non-psychological comparators (three trials, SMD = −1.54, 95% CI (−2.14, −0.94), *p* < 0.001) with low heterogeneity (I^2^ = 15%, *p* = 0.31, for Cochran’s Q test).

### 3.4. Meta-Analysis of Psychological Outcomes

**Figure 4:**
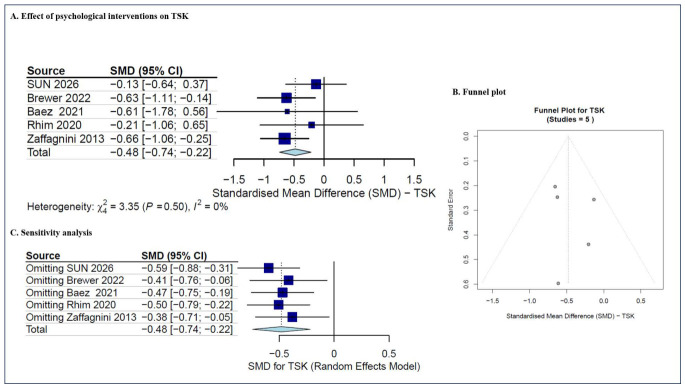
The meta-analysis of TSK [31,32,36,47,50]. (**A**) The effect of psychological interventions on TSK (SMD with 95% CI); (**B**) the funnel plot; and (**C**) the sensitivity analysis.

TSK was reported in five studies [31,32,36,47,50], where a total of 275 patients were included in this research. The pooled data showed a significant difference between intervention and control groups (SMD = −0.48, 95%CI (−0.74, −0.22), *p* < 0.001), with zero heterogeneity (I^2^ = 0%; *p* = 0.50, ns for Cochran’s Q test) (Figure 4A). The predicted 95% CI was −0.79 to −0.16. Our findings demonstrate that psychological interventions yield superior efficacy compared to non-psychosocial control conditions in mitigating kinesiophobia among individuals who have undergone ACLR. Due to the small number of included studies, the funnel plot (Figure 4B) was generated solely for descriptive purposes. The sensitivity analysis remained stable after sequentially removing each study (Figure 4C). These findings indicate that the results are dependable despite the observed heterogeneity.

**Figure 5:**
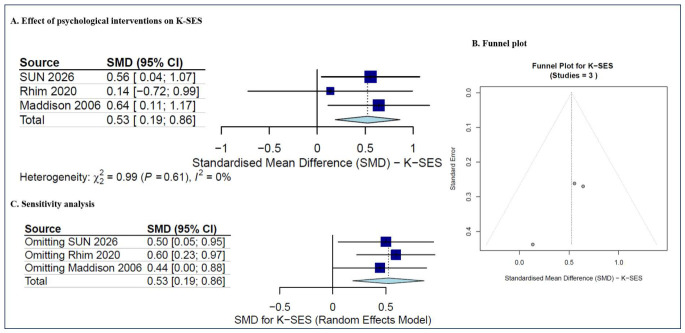
The meta-analysis of K-SES [31,33,50]. (**A**) The effect of psychological interventions on K-SES (SMD with 95% CI); (**B**) the funnel plot; and (**C**) the sensitivity analysis.

K-SES was reported in three studies [31,33,50], where a total of 151 patients were involved in our study. The pooled data showed a significant difference between intervention and control groups (SMD = 0.53, 95%CI (0.19, 0.86), *p* = 0.002), with zero heterogeneity (I^2^ = 0%; *p* = 0.61, ns for Cochran’s Q test) (Figure 5A). The predicted 95% CI was 0.19 to 0.86. The results indicated that psychological intervention improved knee self-efficacy in patients after ACLR more effectively than non-psychosocial comparators. Due to the small number of studies included, the funnel plot (Figure 5B) was generated for descriptive purposes only. The sensitivity analysis revealed no significant changes when each study was removed sequentially (Figure 5C). This suggests the findings are reliable despite heterogeneity.

**Figure 6:**
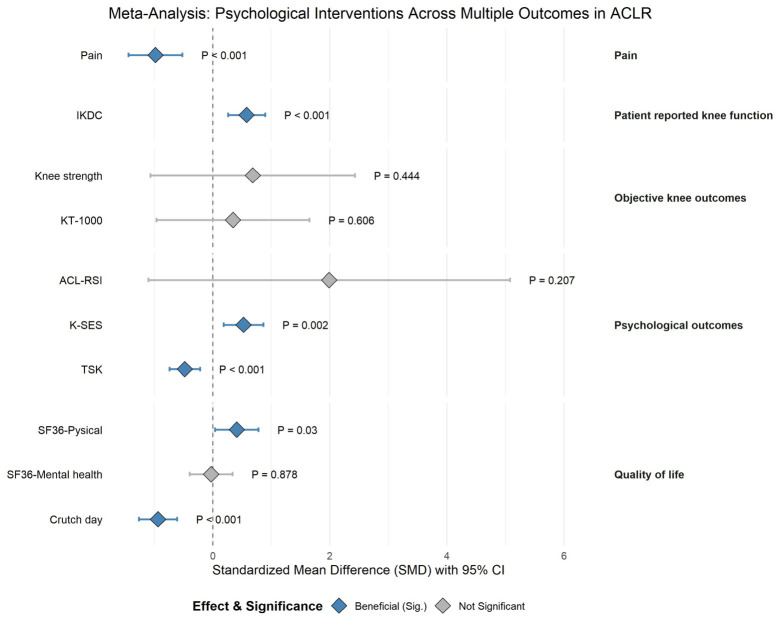
A summary of a meta-analysis examining psychological interventions across multiple outcomes in ACLR (SMD with 95% CI).

ACL-RSI was reported in three studies [31,46,50], where a total of 123 patients were included in the present research. The pooled data revealed no significant difference between intervention and control groups (SMD = 1.98, 95%CI (−1.10, 5.06), *p* = 0.207), with considerable heterogeneity (I^2^ = 94%; *p* = 0.001 for Cochran’s Q test) (Figure 6 and Figure S1). The predicted 95% CI was −4.10 to 8.07. The analysis showed no significant difference, with considerable heterogeneity indicating substantial uncertainty.

However, given the limited sample size of this analysis, future research should incorporate more high-quality, standardized randomized controlled trials to further validate these findings.

### 3.5. Meta-Analysis of Patient-Reported Knee Function

IKDC was reported in two studies involving a total of 159 patients [32,33]. The pooled data showed a significant difference between intervention and control groups (SMD = 0.58, 95%CI (0.26, 0.90), *p* < 0.001), with zero heterogeneity (I^2^ = 0%; *p* = 0.60, ns for Cochran’s Q test). The meta-analysis indicated that psychological intervention was more effective than non-psychosocial comparators in improving IKDC among patients who underwent ACLR (Figure 6 and Figure S2).

Nevertheless, given the limited sample size of this analysis, which included only two studies, future research should incorporate more high-quality, standardized randomized controlled trials to further validate these findings.

### 3.6. Meta-Analysis of Objective Knee Outcomes

KT-1000 was reported in two studies [29,36], and a total of 90 patients were involved in this research. No significant difference was found between the intervention and control groups on KT-1000 (SMD = 0.37, 95% CI (−0.99, 1.72), *p* = 0.606), with substantial heterogeneity (I^2^ = 84.0%; *p* = 0.01 for Cochran’s Q test). See Figure 6 and Figure S3.

Knee strength was reported in two studies [29,30], where a total of 51 patients were involved in the present research. No significant difference was found between the intervention and control groups on knee strength (SMD = 0.68, 95% CI (−1.07, 2.42), *p* = 0.444), with substantial heterogeneity (I^2^ = 85.0%; *p* = 0.01 for Cochran’s Q test). See Figure 6 and Figure S4.

Future studies should incorporate a greater number of high-quality, standardized RCTs to overcome the limitations of small sample sizes and thereby further validate these findings.

### 3.7. Meta-Analysis of Quality of Life

Two studies (125 patients) reported the quality of life measured by 36-item short-form health survey (SF-36) scores before and after ACLR surgery. The random effect meta-analysis standardized mean difference (SMD) for SF-36 is shown in Figure 6 and Figure S5. No significant difference was found between the intervention and control groups on SF-36 mental well-being (SMD = −0.03, 95% CI (−0.39, 0.34), *p* = 0.878), with zero heterogeneity (I^2^ = 0%; *p* = 0.68, ns for Cochran’s Q test). Compared with the control, patients who received psychological intervention were at a significantly greater improvement of physical role function after ACLR (SMD = 0.41, 95% CI (0.04, 0.78), *p* = 0.030), with zero heterogeneity (I^2^ = 0%; *p* = 0.46, ns for Cochran’s Q test).

Crutch-use days were reported in two studies [32,33], where a total of 159 patients were involved in the present research. The pooled data showed a significant difference between intervention and control groups (SMD = −0.94, 95%CI (−1.26, −0.61), *p* < 0.001), with zero heterogeneity (I^2^ = 0%; *p* = 0.35, ns for Cochran’s Q test) (Figure 6 and Figure S6). The results indicated that psychological intervention reduced the number of days patients used crutches after ACLR more effectively than non-psychosocial comparators.

However, because this study included only a small sample, future research should incorporate more high-quality, standardized randomized controlled trials to further verify our findings.

## 4. Discussion

This study provides preliminary evidence for the efficacy of psychological interventions in improving outcomes following ACLR. Our findings demonstrate statistically significant benefits of psychological interventions across multiple domains, including pain reduction, improved psychological outcomes, enhanced patient-reported knee function, and better quality of life. Notably, imagery therapy showed significant efficacy in alleviating postoperative pain. The magnitude of these improvements suggests that psychological interventions are integral components of an accelerated rehabilitation protocol following ACLR.

### 4.1. Pain Management

Our meta-analysis demonstrates a significant reduction in pain severity following ACLR in patients who received psychological interventions. This large effect size suggests that psychological interventions play a crucial role in pain management following ACLR. The mechanisms underlying this improvement may involve reducing pain-catastrophizing thoughts and increasing pain self-efficacy [25,26]. These findings align with previous systematic reviews highlighting the importance of addressing psychological factors in ACLR rehabilitation [27,37].

Several RCTs have shown mixed results regarding specific psychological interventions’ impact on pain. While Brewer et al. [36] and Zaffagnini et al. [32] demonstrated significant pain reductions with cognitive therapy. Maddison et al. [29] found non-significant effects with techniques like imagery therapy. Similarly, Cupal [30] and Lebon [48] reported pain reduction in imagery therapy, but the effect was not statistically significant at all time points. These inconsistencies underscore the need for comprehensive meta-analyses. Based on our findings, rehabilitation protocols could incorporate psychological interventions for pain management in clinical practice. However, the specific management strategies require further investigation to be determined.

### 4.2. Various Types of Psychological Intervention

Among the six trials reporting pain severity, three studies employed imagery therapy, highlighting its potential advantages in ACLR rehabilitation.

While the exact mechanisms remain to be fully elucidated, potential pathways such as enhanced cognitive engagement, promoting neuroplasticity, and motor learning [51], are hypothesized based on previous research, but require further direct investigation; secondly, they often incorporate relaxation elements, which can help reduce stress and anxiety associated with injury and rehabilitation, thereby modulating pain perception [30]; moreover, by mentally rehearsing successful movement and recovery, patients may experience increased self-efficacy and confidence in managing pain [29].

While imagery therapy demonstrated significant efficacy in our analysis, other approaches also show promise. Cognitive-behavioral therapy (CBT) [36] has been reported to improve pain and function, while exposure therapy [47] and modeling videos [33] have also shown positive effects. Positive self-talk therapy [27] may foster a more optimistic outlook, increasing patients’ motivation to engage in their recovery process, and goal-setting interventions [34] can enhance motivation and exercise adherence. Counseling [26] provides social support, which can offer emotional validation and practical guidance, helping patients navigate the challenges of recovery, offering emotional validation and practical guidance.

Future research should aim to directly compare different psychological interventions, explore potential synergies between techniques, and identify patient characteristics that predict better responses to specific interventions.

### 4.3. Psychological Outcomes

The small effect size (SMD = −0.48) and absence of heterogeneity (I^2^ = 0%) in TSK scores underscore the consistency and effectiveness of psychological interventions in reducing fear of movement post-ACLR. This finding extends previous research by Aynollah et al. [39], providing preliminary evidence with a larger sample size, broader outcome measure, and lower heterogeneity. Mechanisms underlying this improvement may include cognitive restructuring [52], exposure therapy [53], and observational learning [54]. Reduced kinesiophobia can contribute to better functional outcomes, as patients may be more willing to challenge themselves during rehabilitation [12] and may have positive implications for overall psychological well-being, potentially reducing anxiety and depression associated with injury and surgery [55].

This study also reveals a moderate effect on improving knee self-efficacy (K-SES: 3 trials, SMD = 0.53, 95% CI 0.19–0.86, I^2^ = 0%). Self-efficacy directly affects patients’ rehabilitation motivation [19]. Improved knee self-efficacy helps enhance patients’ confidence in rehabilitation and improve training adherence [50]. In clinical practice, personalized small-goal rehabilitation plans can be developed for patients with low self-efficacy to strengthen self-awareness through phased success.

However, no significant effect was observed on ACL-RSI (*p* > 0.05), and there was substantial heterogeneity. ACL-RSI assesses patients’ confidence and actual behavior in returning to sports, which is affected by multiple factors such as objective knee function level, social support, and the type of exercise [56]. The lack of significance in ACL-RSI results may be related to the heterogeneity of the included studies, as different studies used different intervention methods and assessment time points. Future studies should adopt standardized intervention protocols and unified assessment tools to more accurately evaluate the effect of psychological interventions on ACL-RSI.

### 4.4. Patient-Reported Knee Function

In this study, psychological interventions moderately improved patient-reported knee function (IKDC, SMD = 0.58, 95% CI 0.26 to 0.90, I^2^ = 0%). The potential mechanism may be that psychological interventions indirectly improve patients’ adherence to rehabilitation training by reducing pain, alleviating kinesiophobia, and enhancing self-efficacy, thereby promoting knee function recovery. Unlike objective function indicators, IKDC focuses more on patients’ subjective perception of function, and improvement in psychological status can significantly enhance patients’ subjective satisfaction with knee function [57]. This suggests that in post-ACLR rehabilitation, we should not only focus on objective function indicators but also pay attention to patients’ subjective feelings. In clinical practice, psychological interventions could be combined with physical therapy to maximize patients’ functional recovery.

### 4.5. Objective Knee Outcomes

Our meta-analysis found no significant improvement in KT-1000 (knee stability measurement) or knee muscle strength with psychological interventions. KT-1000 (reflecting anterior–posterior knee laxity [58]) and knee muscle strength mainly reflect the physiological structure and muscle function of the knee joint, which are more affected by factors such as surgical techniques and the intensity of physical rehabilitation training. Psychological interventions mainly affect the rehabilitation process indirectly by regulating the psychological status, with limited direct effects on objective physiological indicators. In clinical practice, it should be clear that psychological interventions cannot replace physical rehabilitation training in improving objective knee outcomes. Psychological interventions need to be combined with standardized physical rehabilitation programs, paying attention to both patients’ psychological status and the intensity and standardization of physical training.

### 4.6. Quality of Life

The effectiveness of psychological interventions on quality of life in patients following ACLR is inconsistent. We found a small, significant improvement on the physical role function dimension of the SF-36, but no significant difference in the mental well-being dimension. Additionally, psychological interventions significantly reduced the number of days patients used crutches after ACLR. Additionally, crutch-use days, as a critical extension indicator of quality of life, not only reflect the objective recovery of lower limb function but also underscore the important role of psychological interventions in improving patients’ independent living abilities. Being free from crutches allows patients to engage more freely in daily activities, reduces reliance on others, and thereby significantly improves their quality of life [59]. The results of this study indicate that psychological interventions significantly reduced the number of days patients used crutches after ACLR, with a large effect size.

The improvement in physical role function and the reduction in days using crutches may be explained by several potential factors. Psychological interventions may reduce fear of movement, leading to increased involvement in physical activities [60]. Better pain management could allow patients to participate more fully in rehabilitation exercises and daily activities [52]. Additionally, interventions like modeling videos may enhance patients’ confidence in performing physical tasks [32], while addressing negative thought patterns about physical capabilities could promote a more positive attitude to recovery [37]. The absence of significant improvement in mental well-being is intriguing. Possible explanations include high baseline mental well-being levels [61], delayed mental health improvements beyond the study timeframes [12], interventions focusing more on physical function and pain rather than general mental well-being [32], the SF-36 mental well-being dimension’s potential lack of sensitivity to ACLR-specific changes [18], or the small sample size and short intervention duration of included studies. Our findings highlight the potential of psychological interventions to enhance physical role function in ACLR patients, while also revealing the complexity of addressing mental well-being. Future research should focus on developing more comprehensive and personalized psychological interventions that effectively target both physical and mental health outcomes in ACLR rehabilitation. In clinical practice, psychological interventions can be combined with physical therapy to help patients improve their daily activities.

### 4.7. Limitations

Several limitations should be noted. First, the small number of included studies and relatively small total sample size—which also means publication bias could not be reliably assessed—combined with an overall high risk of bias across studies (two with some concerns, and two with high risk), limit the generalizability and reliability of the findings, highlighting the need for more large-scale, high-quality randomized controlled trials in this area. Second, there was significant heterogeneity in the types of psychological interventions used across studies, ranging from cognitive-behavioral therapy to mindfulness-based approaches, and a lack of standardization in intervention duration, frequency, and intensity, which complicates the identification of specific psychological methods that yield the best outcomes; future research should aim to compare different psychological intervention modalities to identify the most beneficial approaches. Third, the lack of standardization in outcome measures across studies makes direct comparisons challenging, even though we were able to pool data for some outcomes; future research would benefit from the adoption of standardized, validated outcome measures specific to anterior cruciate ligament reconstruction (ACLR) patients. Fourth, the potential language bias resulting from the English-only inclusion may have omitted relevant studies published in other languages; future research should consider including multilingual literature to improve the generalizability of findings. Additionally, the short follow-up duration (≤6 months) of included studies means there is an absence of long-term outcome data, preventing a comprehensive understanding of the sustained efficacy of psychological interventions; future research should extend follow-up periods to assess long-term effects. Furthermore, there is a limited exploration of return-to-sport outcomes, which are highly relevant for ACLR patients and represent a key rehabilitation goal that warrants greater attention in future studies to more fully understand the role of psychological interventions in patient recovery. Despite these limitations, this study provides preliminary evidence for the application of psychological interventions in post-ACLR rehabilitation and identifies important directions for future research.

## 5. Conclusions

In conclusion, this meta-analysis provides preliminary evidence for the potential effectiveness of psychological interventions in ACLR rehabilitation, demonstrating improvements in pain, psychological outcomes, patient-reported knee function, and quality of life. Notably, imagery therapy shows promise in pain management following ACLR. Future high-quality, adequately powered RCTs are needed to confirm these preliminary findings and to establish whether specific psychological strategies can be effectively standardized and implemented in ACLR rehabilitation protocols.

## Figures and Tables

**Table 1:** Summary of reviewed studies.

Authors (Years)	Country	Randomized, Age, Male	Psychological Intervention	Control Intervention	Timing of Intervention	Follow-Up Times	Outcome Measures	Funding Sources
Baez (2021) [47]	USA	N = 12 (6:6), 22.5 ± 4.6 years, 0	Exposure therapy	Usual intervention	Post surgery	2 (pre and post intervention)	Psychological outcomes (PHOSA-ACLR, TSK); patient-reported knee function (KOOS)	National Athletic Trainers’ Association Research & Education Foundation
Brewer (2022) [36]	USA	N = 69 (34:35), 35.01 ± 11.98 years, 39	Cognitive-behavioral therapy	Placebo intervention	Pre and Post surgery	7 (pre and post 1, 2, 3, 4, 5, 6 months)	Pain (NRS), psychological outcomes (TSK); patient-reported knee function (KOS-SAS); objective knee outcomes (ROM, KT-1000)	National Institute of Arthritis and Musculoskeletal and Skin Diseases
Cappellino (2012) [49]	Italy	N = 14 (7:7), 27.5 ± 5.1 years, 14	Neurocognitive interventions	Conventional exercises	Post surgery	4 (pre and post 1, 3, 6 months)	Pain (VAS); objective knee outcomes (ROM); quality of life (SF-36)	NA
Cupal (2001) [30]	USA	N = 30 (10:10:10), 38.2 ± 8.2 years, 16	Relaxation and guided imagery	Placebo intervention	Post surgery	2 (pre and post intervention)	Pain (VAS); objective knee outcomes (knee strength)	NA
D’Isanto (2022) [46]	Italy	N = 30 (15:15), 32.5 ± 1.8 years, 0	Guided imagery and mirror therapy	Usual intervention	Post surgery	2 (pre and post)	Pain (VAS); psychological outcomes (ACL-RSI)	This research received no external funding
Lebon (2012) [48]	New Zealand	N = 12 (7:5), 28.5 ± 5.0 years, 10	Motor imagery	Usual intervention	Post surgery	2 (pre and post)	Pain (VAS); patient- reported knee function (LEFS); objective knee outcomes (EMG, ROM)	NA
Maddison (2012) [29]	New Zealand	N = 21 (13:8), 34.86 ± 8.84 years, 13	Relaxation and guided imagery	Placebo intervention	Post surgery	5 (pre, during 2, 6, 12 weeks, and post)	Objective knee outcomes (knee strength, KT-1000)	NA
Maddison (2006) [33]	New Zealand	N = 58 (30:28), 15–53 years, 39	Modeling videos	Usual intervention	Pre surgery	3, (pre, post, and post 2 weeks)	Pain (NRS); patient-reported knee function (IKDC, ROM); psychological outcomes (STAI, K-SES); quality of life (crutch-use days)	NA
Rhim (2020) [31]	Korea	N = 32 (10:11:11), 28.8 ± 10.5 years, 27	Modeling videos	Placebo videos	Post surgery	6 (pre, during 0, 2, 6 weeks, and post	Psychological outcomes (TSK-11, ACL-RSI, K-SES); patient-reported knee function (KOOS)	National Research Foundation of Korea Grant
Zaffagnini (2013) [32]	Italy	N = 101 (51:50), 33.4 ± 11.8 years, 21	Modeling videos	Placebo videos	Post surgery	2 (pre and post)	Patient-reported knee function (IKDC); psychological outcomes (TSK); quality of life (SF-36, crutch-use days)	There was neither a financial nor a personal relationship that could inappropriately influence their work
Sun (2026) [50]	China	N = 61 (29:32), 28.5 ± 7.0 years, 41	Cognitive-behavior-based physical therapy	Usual intervention	Pre and Post surgery	4 (pre and post 6, 12, 24 weeks)	Psychological outcomes (ACL-RSI, TSK, K-SES, BRS, LOC); patient- reported knee function (Lysholm score)	Fujian Provincial Finance Department

Note: PHOSA-ACLR: Photographic Series of Sports Activities for ACLR, TSK: Tampa Scale of Kinesiophobia, KOOS: Knee Injury and Osteoarthritis Outcome Score, NRS: Numerical Rating Scale, KOS-SAS: Knee Outcomes Survey–Sports Activities Scale, VAS: Visual Analog Scale, SF-36: 36-item short-form health survey, ROM: range of motion, ACL-RSI: Anterior Cruciate Ligament–Return to Sport After Injury questionnaire, EMG: electromyographic, LEFS: Lower Extremity Functional Scale, IKDC: the International Knee Documentation Committee system, STAI: the State–Trait Anxiety Inventory, K-SES: Knee Self-Efficacy Scale, BRS: Brief Resilience Scale, LOC: Locus of Control.

**Table 2:** Risk of bias (RoB 2) and certainty of evidence (GRADE) summary for the included studies.

Study	RoB 2 Overall Risk	Key Sources of Bias	GRADE Certainty	Reasons for Downgrading
Sun (2026) [50]	Low risk	Unclear allocation concealment; imbalanced attrition	Moderate	Imprecision (−1)
D’Isanto (2022) [46]	Low risk	Randomization details missing; subjective outcomes	Low	Imprecision (−1); Indirectness (−1)
Brewer (2022) [36]	High risk	High attrition (31%); lack of blinding	Very low	Risk of bias (−1); Imprecision (−2); Indirectness (−1)
Baez (2021) [47]	Some concerns	Subjective outcomes; acknowledged reporting bias	Very low	Risk of bias (−1); Imprecision (−2); Indirectness (−1)
Rhim (2020) [31]	Low risk	Participants unblinded; adherence not monitored	Low	Imprecision (−1)
Zaffagnini (2013) [32]	High risk	High and imbalanced attrition; incomplete outcome reporting	Low	Risk of bias (−1); Imprecision (−1)
Maddison (2012) [29]	Some concerns	High missing data rate; small sample size	Low	Risk of bias (−1); Imprecision (−1)
Lebon (2012) [48]	Low risk	Randomization details missing	Moderate	Imprecision (−1)
Cappellino (2012) [49]	Low risk	Subjective outcome measures	Moderate	Imprecision (−1)
Maddison (2006) [33]	Low risk	Subjective outcome measures	Moderate	Imprecision (−1)
Cupal (2001) [30]	Low risk	Subjective outcome measures	Moderate	Imprecision (−1)

Note: RoB 2: Cochrane risk of bias tool version 2 and GRADE: Grading of Recommendations Assessment, Development and Evaluation.

## Data Availability

The datasets used and/or analyzed during the current study are available from the corresponding author upon reasonable request following article publication. All data used in this meta-analysis were extracted from previously published articles.

## Supplementary Materials

The following supporting information can be downloaded at https://www.mdpi.com/article/10.3390/jcm15103980/s1, Supplementary Material S1: PRISMA 2020 Checklist [62]; Supplementary Material S2: Search strategies and Supplementary figures; Figure S1: Meta-analysis of ACI-RSI. (A) Effect of psychological interventions on ACL-RSI (SMD with 95% CI); (B) Funnel plot; Figure S2: Effect of psychological interventions on IKDC; Figure S3: Effect of psychological interventions on KT-1000; Figure S4: Effect of psychological interventions on Knee strength; Figure S5: Meta-analysis of quality of life. (A) Effect of psychological interventions on SF36-Mental health (SMD with 95% CI); (B) Effect of psychological interventions on SF36-Pysical (SMD with 95% CI); Figure S6: Effect of psychological interventions on Crutch day.
