# Supplementary material for: The Effectiveness of Psychological Interventions for Patients Undergoing Anterior Cruciate Ligament Reconstruction: A Meta-Analysis and Systematic Review
Source: J Clin Med. 2026 May 21;15(10):3980. doi: 10.3390/jcm15103980 (PMC13208142; doi:10.3390/jcm15103980)
Supplement: Supplementary file 1 [file jcm-15-03980-s001.zip › jcm-4234194 Supplementary Material S2.pdf]

Supplementary Material S2

1. Search strategies

1) PubMed

Search Strategy:

1	Anterior Cruciate Ligament [MeSH Terms]
2	Anterior Cruciate Ligament Injuries [MeSH Terms]
3	Anterior Cruciate Ligament Reconstruction [MeSH Terms]
4	Anterior Cruciate Ligament Tear [MeSH Terms]
5	ACL Injuries
6	ACL Tears
7	1 or 2 or 3 or 4 or 5 or 6
8	Psychotherapy [MeSH Terms]
9	Art Therapy
10	Behavior Therapy
11	Cognitive Behavioral Therapy
12	Relaxation Therapy
13	Crisis Intervention
14	Dance Therapy
15	Emotion-Focused Therapy
16	Guided Imagery
17	Mentalization-Based Therapy
18	Music Therapy
19	Psychosocial Intervention
20	Psychotherapeutic Processes
21	Socioenvironmental Therapy
22	Group Psychotherapy
23	8 or 9 or 10 or 11 or 12 or 13 or 14 or 15 or 16 or 17 or 18 or 19 or 20 or 21 or 22
24	7 AND 23

2) MEDLINE

Database: Ovid MEDLINE(R) ALL <1946 to July 19, 2024>

Search Strategy:

1	exp Anterior Cruciate Ligament Reconstruction/
2	exp Anterior Cruciate Ligament Injuries/
3	exp Anterior Cruciate Ligament/
4	ACL Injuries.mp. 2
5	ACL Injury.mp.
6	ACL Tears.mp.
7	Anterior Cruciate Ligament Tear.mp
8	ACL Tear.mp
9	1 or 2 or 3 or 4 or 5 or 6 or 7 or 8
10	exp Psychotherapy/
11	exp Art Therapy/
12	exp Behavior Therapy/

13	exp Cognitive Behavioral Therapy/
14	exp Relaxation Therapy/
15	exp Crisis Intervention/
16	exp Dance Therapy/
17	exp Emotion-Focused Therapy/
18	exp Imagery, Psychotherapy/
19	exp Mentalization-Based Therapy/
20	exp Music Therapy/
21	exp Psychosocial Intervention/
22	exp Psychotherapeutic Processes/
23	exp Socioenvironmental Therapy/
24	exp Psychotherapy, Group/
25	exp Guided Imagery/
26	10 or 11 or 12 or 13 or 14 or 15 or 16 or 17 or 18 or 19 or 20 or 21 or 22 or 23 or 24 or 25
27	9 and 26

3) Embase

Database: Embase <1974 to 2021 July 19>

Search Strategy

#1	'anterior cruciate ligament'
#2	'anterior cruciate ligament injuries'
#3	'acl injuries'
#4	'acl injury'
#5	'anterior cruciate ligament tear'
#6	'acl tears'
#7	'anterior cruciate ligament reconstruction'
#8	#1 OR #2 OR #3 OR #4 OR #5 OR #6 OR #7
#9	'psychotherapy'
#10	'art therapy'
#11	'behavior therapy'
#12	'cognitive behavioral therapy'
#13	'relaxation therapy'
#14	'crisis intervention'
#15	'dance therapy'
#16	'emotion-focused therapy'
#17	'guided imagery'
#18	'mentalization-based therapy'
#19	'music therapy'
#20	'psychosocial intervention'
#21	'psychotherapeutic processes'
#22	'socioenvironmental therapy'
#23	'group psychotherapy'

#24	#9 OR #10 OR #11 OR #12 OR #13 OR #14 OR #15 OR #16 OR #17 OR #18 OR #19 OR #20 OR #21 OR #22 OR #23
#25	#8 AND #24

4) Cochrane Library

Search Strategy

#1	‘Anterior Cruciate Ligament Reconstruction’
#2	‘Anterior Cruciate Ligament’
#3	‘Anterior Cruciate Ligament Injuries’
#4	‘ACL Injuries’
#5	‘Anterior Cruciate Ligament Tears’
#6	‘ACL Tears’
#7	#1 OR #2 OR #3 OR #4 OR #5 OR #6
#8	'Psychotherapy'
#9	'Art Therapy'
#10	'Behavior Therapy'
#11	'Cognitive Behavioral Therapy'
#12	'Relaxation Therapy'
#13	'Crisis Intervention'
#14	'Dance Therapy'
#15	'Emotion-Focused Therapy'
#16	'Guided Imagery'
#17	'Mentalization-Based Therapy'
#18	'Music Therapy'
#19	'Psychosocial Intervention'
#20	'Psychotherapeutic Processes'
#21	'Socioenvironmental Therapy'
#22	'Group Psychotherapy'
#23	#8 OR #9 OR #10 OR #11 OR #12 OR #13 OR #14 OR #15 OR #16 OR #17 OR #18 OR #19 OR #20 OR #21 OR #22
#24	#7 AND #23

5) PsycInfo

Search Strategy

S1	DE "Knee"
S2	TI ("Knee Pain" or " Anterior Cruciate Ligament Injuries " or " ACL Injuries " or " ACL Tears ") OR AB ("Knee Pain" or " Anterior Cruciate Ligament Injuries " or " ACL Injuries " or " ACL Tears ")
S3	TI (knee# N (Anterior Cruciate Ligament Reconstruction* or ACLR* or disease# or pain#) OR AB (knee# N (Anterior Cruciate Ligament Reconstruction * or ACLR * or disease# or pain#)
S4	TI ((radiographic* or symptomatic* or clinical*) N1 " Anterior Cruciate Ligament Reconstruction*") OR AB ((radiographic* or symptomatic* or

clinical*) N1 " Anterior Cruciate Ligament Reconstruction *")

S5 S1 OR S2 OR S3 OR S4

S6 ((((((((DE "Psychotherapy") OR (DE "Psychology")) OR (DE " Psychosocial Intervention ")) OR (DE "Social Psychology")) OR (DE "Cognitive Behavior Therapy")) OR (DE "Relaxation Therapy")) OR (DE "Relaxation")) OR (DE "Imagery")) OR (DE "Hypnosis")) OR (DE "Behavior Therapy" OR DE " Art Therapy " OR DE " Crisis Intervention " OR DE " Emotion-Focused Therapy " OR DE "Exposure Therapy" OR DE " Mentalization-Based Therapy " OR DE " Music Therapy " OR DE " Group Psychotherapy " OR DE " Socioenvironmental Therapy ")

S7 TI ((psychotherap* or psychological or psychology or psychoeducation or "cognitive therap*" or "behavio#ral therap*" or relaxation or imagery or hypnosis or psychosocial or (adapt* N behavio#r#) or (behavio#r# N (therap* or intervention*)))) OR AB ((psychotherap* or psychological or psychology or psychoeducation or "cognitive therap*" or "behavio#ral therap*" or relaxation or imagery or hypnosis or psychosocial or (adapt* N behavio#r#) or (behavio#r# N (therap* or intervention*))))

S8 S6 OR S7

S9 (((DE "Treatment Effectiveness Evaluation") OR (DE "Treatment Outcomes" OR DE "Psychotherapeutic Outcomes" OR DE "Side Effects (Treatment)" OR DE "Treatment Compliance" OR DE "Treatment Duration" OR DE "Treatment Refusal" OR DE "Treatment Termination" OR DE "Treatment Withholding")) OR (DE "Placebo")) OR (DE "Followup Studies")

S10 TX placebo* OR random* OR "comparative stud*" OR clinical N3 trial* OR research N3 design OR evaluat* N3 stud* OR prospectiv* N3 stud* OR (singl* OR doubl* OR trebl* OR tripl*) N3 (blind* OR mask*)

S11 S9 OR S10

S12 S8 AND S11

S13 S5 AND S12

2. Supplementary figures

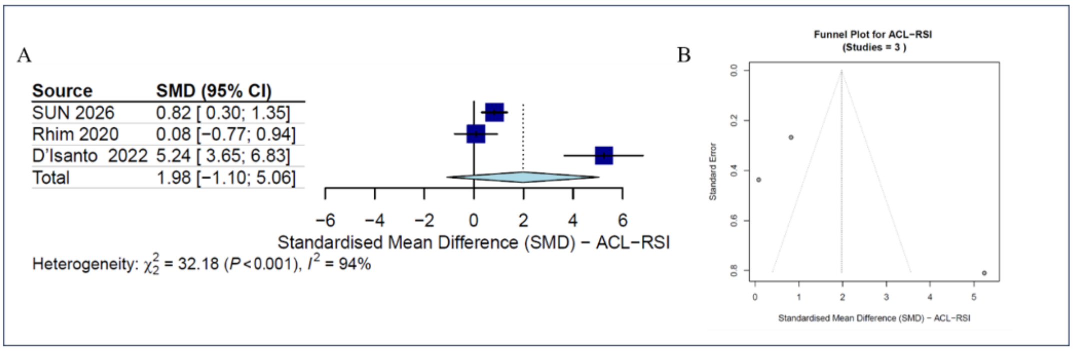

Figure S1. The Meta-analysis of ACI-RSI. (A) The effect of psychological interventions on ACL-RSI (SMD with 95% CI); and (B) the funnel plot.

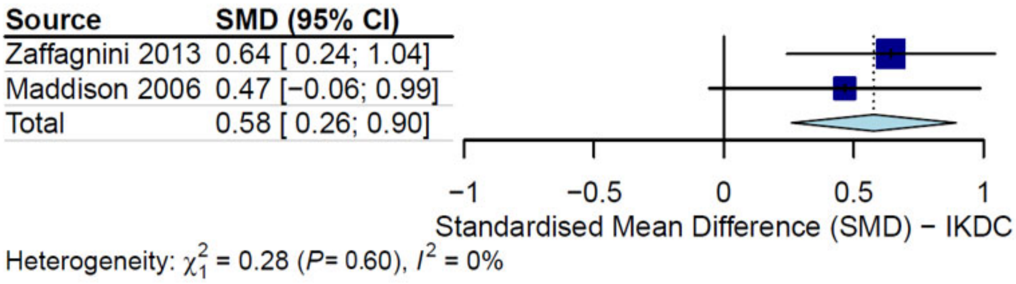

Figure S2. Effect of psychological interventions on IKDC

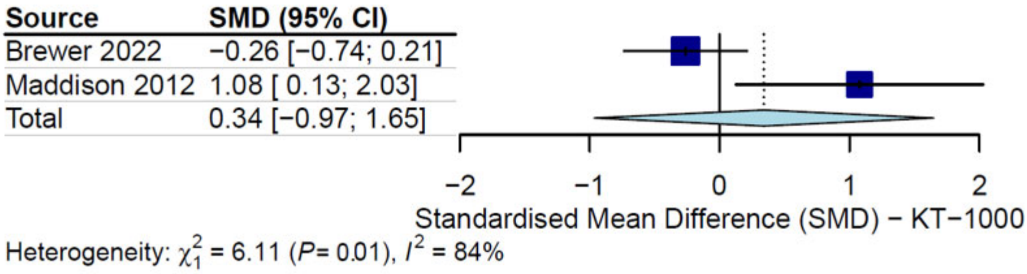

Figure S3. Effect of psychological interventions on KT-1000

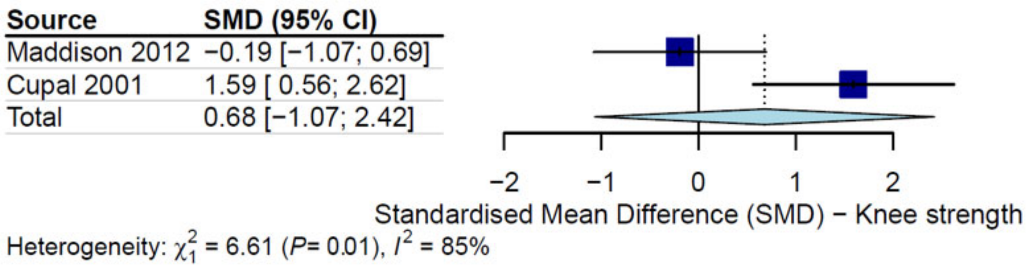

Figure S4. Effect of psychological interventions on Knee strength

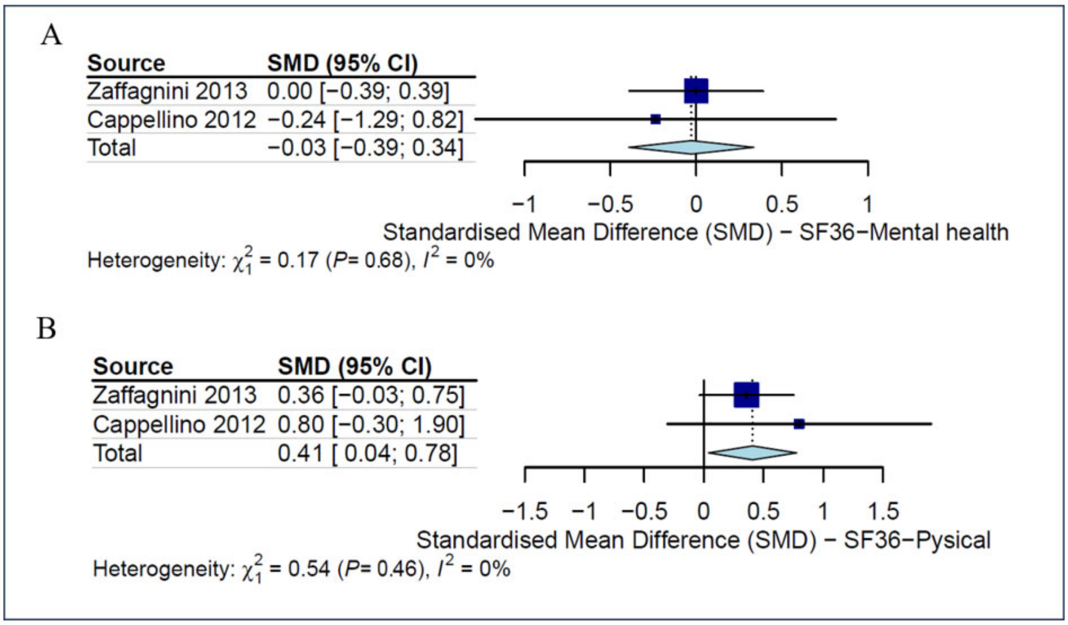

Figure S5. The Meta-analysis of quality of life. (A) The effect of psychological interventions on SF36-Mental health (SMD with 95% CI); and (B) the effect of psychological interventions on SF36-Pysical (SMD with 95% CI).

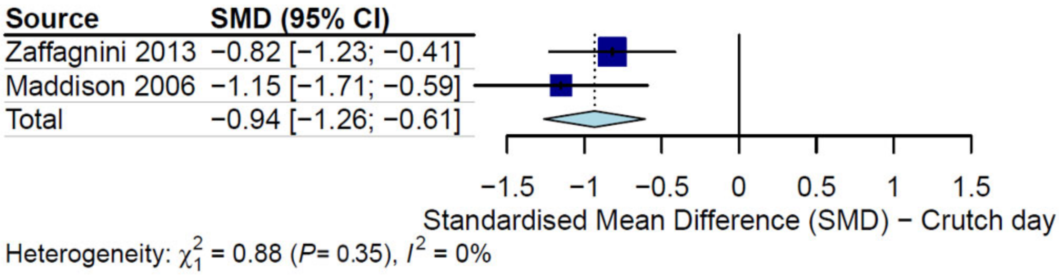

Figure S6. Effect of psychological interventions on Crutch day.
